# Erythritol Is More Effective Than Xylitol and Sorbitol in Managing Oral Health Endpoints

**DOI:** 10.1155/2016/9868421

**Published:** 2016-08-21

**Authors:** Peter de Cock, Kauko Mäkinen, Eino Honkala, Mare Saag, Elke Kennepohl, Alex Eapen

**Affiliations:** ^1^Cargill R&D Centre Europe, Havenstraat 84, 1800 Vilvoorde, Belgium; ^2^Institute of Dentistry, University of Turku, Lemminkäisenkatu 2, 20520 Turku, Finland; ^3^Institute of Dentistry, Faculty of Medicine, University of Tartu, Raekoja Plats 6, 51003 Tartu, Estonia; ^4^Equinox Scientific Services, 11-26520 Twp Road 512, Spruce Grove, AB, Canada T7Y 1G1; ^5^Cargill Incorporated, 15407 McGinty Road West (MS-163), Wayzata, MN 55391, USA

## Abstract

*Objective*. To provide a comprehensive overview of published evidence on the impact of erythritol, a noncaloric polyol bulk sweetener, on oral health.* Methods*. A literature review was conducted regarding the potential effects of erythritol on dental plaque (biofilm), dental caries, and periodontal therapy. The efficacy of erythritol on oral health was compared with xylitol and sorbitol.* Results*. Erythritol effectively decreased weight of dental plaque and adherence of common streptococcal oral bacteria to tooth surfaces, inhibited growth and activity of associated bacteria like* S. mutans*, decreased expression of bacterial genes involved in sucrose metabolism, reduced the overall number of dental caries, and served as a suitable matrix for subgingival air-polishing to replace traditional root scaling.* Conclusions*. Important differences were reported in the effect of individual polyols on oral health. The current review provides evidence demonstrating better efficacy of erythritol compared to sorbitol and xylitol to maintain and improve oral health.

## 1. Introduction

Erythritol is a four-carbon polyol (sugar alcohol) that shares many of the functional properties that are typical for the polyols family (e.g., sorbitol, xylitol). Such characteristics are important in practical applications of erythritol and include the following general features: relatively high stability in acidic and alkaline environments, high stability against heat, sweetness close to that of sucrose, calorific reduction compared to sucrose, safety, no cariogenic potential, low glycemic index, and suitability as a bulking agent in food manufacturing [1]. Erythritol differentiates from all other polyols in that it is commercially produced using fermentation, a recognized natural process. Erythritol has been reported to have approximately 60–80% of the sweetness of sugar [2, 3], while contributing no calories (noncaloric) and having good digestibility (well tolerated) without any impact on blood glucose and insulin levels [4].

A significant volume of toxicology and safety studies exists, showing a complete lack of adverse effects associated with consumption of erythritol [5]. This body of evidence demonstrating the safety of erythritol, combined with its zero-calorie sweetness and mouth feel, is all key contributors to the general acceptance by consumers for use in a wide variety of food products [5, 6].

While known for its nutritional and technological benefits, erythritol has also been shown to exert a number of beneficial oral health effects (summarized in Table 1). Specifically, the noncariogenicity of erythritol was established first in rats by a group of researchers in 1992 [7].

Inclusion of erythritol in studies aimed at investigating the effects of polyols on dental caries followed the logical scientific process: other common polyols, notably xylitol and sorbitol, both of which are commonly used as sugar replacers in food products, had for dozens of years been studied as potential caries-preventive agents. Inventions related to erythritol manufacturing and comprehensive safety conclusions of the metabolic effects of erythritol have marked its gradual advent in the real world of consumers. The palette of polyol sweeteners has been expanding and may be nearing completion, since the number of physiologically acceptable polyol sweeteners can be considered limited. Erythritol may be regarded as a welcome addition to this palette.

The present review deals with erythritol primarily from the oral health perspective.

## 2. Regulatory Status of Erythritol

Erythritol has a significant history of safe use, with products being consumed in the marketplace since 1990 after being authorized for use in foods in many countries including Japan, USA, Canada, Brazil, Argentina, Paraguay, Uruguay, China, India, Israel, Mexico, Philippines, Singapore, Australia, New Zealand, South-Korea, Taiwan, and Thailand. Erythritol is currently approved for use in foods and beverages and marketed in more than 60 countries around the world [18]. Euromonitor [19] has published consumption levels of erythritol from 2007 through 2012. The results show that on a worldwide basis the consumption levels of erythritol in 2012 accounted for approximately 25,500 metric tons and this level had been increasing steadily throughout this time period. This is highlighted specifically in the United States, which is the major consumer of erythritol, accounting for more than 13,000 metric tons in 2012 having grown from a value of 1,800 metric tons in 2007 (data from Euromonitor [19]).

## 3. Safety of Erythritol

A thorough safety evaluation of erythritol appeared in October 1996 in* Regulatory Toxicology and Pharmacology* (Volume 24, Number 2). The fifteen publications [4, 20–33] included in this safety review of erythritol demonstrate that erythritol is well tolerated and does not cause any toxicologically relevant effects even after ingestion of larger quantities. The digestive tolerance studies of Tetzloff et al. [24] confirmed the safety of oral erythritol: repeated ingestion of erythritol at daily doses of 1 g/kg body weight was well tolerated by humans. Even when consumed under most severe conditions as a single bolus dose in a beverage within 15 minutes on an empty stomach, an erythritol dose of 0.7 g/kg body weight did not cause laxation in adults [34] nor in young (4–6 years of age) children [35]. In comparison, the dose at which xylitol did not show a laxative effect in adults under the same severe conditions was about 0.3 g/kg bw [34]. It is anticipated that the exposure to erythritol* via* oral healthcare products will be very low at approximately 0.1 g/kg body weight per day, which is only 10% of the reported well-tolerated dose in humans [24].

## 4. Erythritol Suppresses Harmful Bacteria and Reduces Biofilm (Dental Plaque)

Erythritol was investigated for its potential to inhibit the growth of bacteria in dental plaque (i.e., a biofilm of microbial accumulations, particularly* S. mutans*) [8–11, 13, 14, 16, 17], as summarized in Table 1 and described below.

Mäkinen et al. [8, 9] conducted 2 preliminary xylitol, erythritol, and sorbitol comparison studies looking at their effects on saliva and plaque levels of* S. mutans*. In the first study [8], 2 groups of 15 subjects (mean age of 30.3 ± 17.1 years) were given either xylitol- or erythritol-containing chewable tablets (10 tablets/day) for a period of 2 months with a daily polyol intake of 5.2 g. Xylitol, but not erythritol, showed a statistically significant reduction of dental plaque and saliva and plaque levels of* S. mutans*. In the second study [9], subjects also were given chewable tablets (10 tablets/day) that contained xylitol (*n* = 26), sorbitol (*n* = 24), xylitol-erythritol (*n* = 22), or sorbitol-erythritol (*n* = 23) for up to 64 days. Total daily polyol consumption was 5.4 g/day (mixtures contained 2.7 g/day of each polyol). A significant reduction in plaque and saliva counts of* S. mutans* was demonstrated for xylitol alone and for the 1 : 1 xylitol mixture with erythritol. Since erythritol was not administered alone, it is not possible to determine erythritol's contribution to this effect. However, the relative portion of* S. mutans* of total streptococci at endpoint was significantly higher in the sorbitol group compared with the sorbitol-erythritol group (*p* = 0.007). In fact, the relative portion of* S. mutans* of total streptococci at endpoint was at the same low level in the sorbitol-erythritol group as in the xylitol-erythritol group indicating a strong contribution of erythritol in the* S. mutans*-reducing effects.

**Figure 1:**
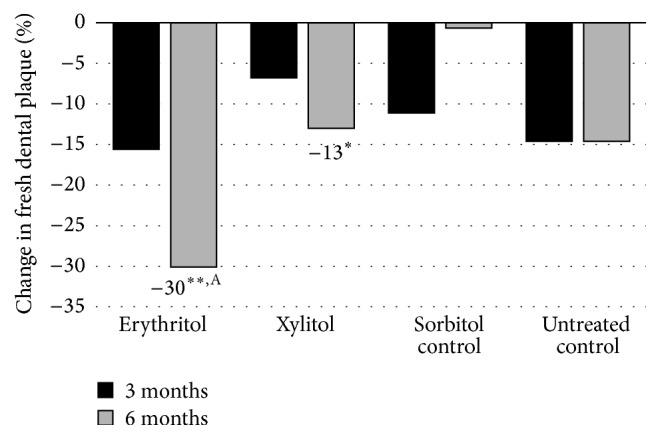
Percent change in fresh dental plaque weight against baseline over a 6-month period in a teenage cohort consuming erythritol-, sorbitol-, or xylitol-containing chewable tablets. Adapted from Mäkinen et al. [10] and Mäkinen (personal communication). ^*∗*^ *p* < 0.05 when compared to baseline using a paired *t*-test. ^*∗∗*^ *p* < 0.001 when compared to baseline using a paired *t*-test. ^A^ *p* < 0.05 changes from baseline when compared with untreated control, sorbitol, or xylitol.

**Figure 2:**
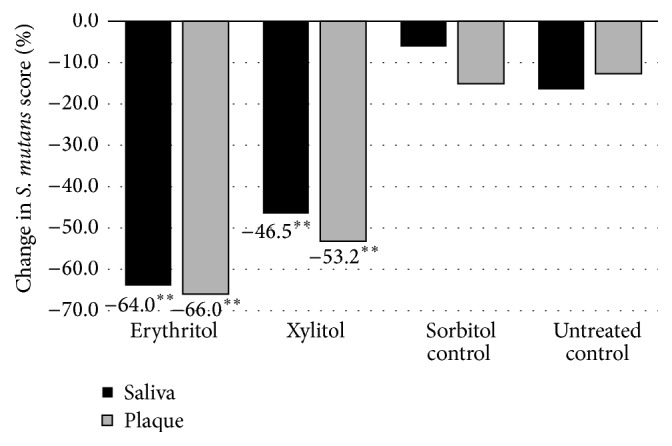
Percent change in salivary and plaque* S. mutans* score against baseline over a 6-month period in a teenage cohort consuming erythritol-, sorbitol-, or xylitol-containing chewable tablets. Adapted from Mäkinen et al. [10]. ^*∗∗*^ *p* < 0.001 when compared to baseline using a paired *t*-test.

**Figure 3:**
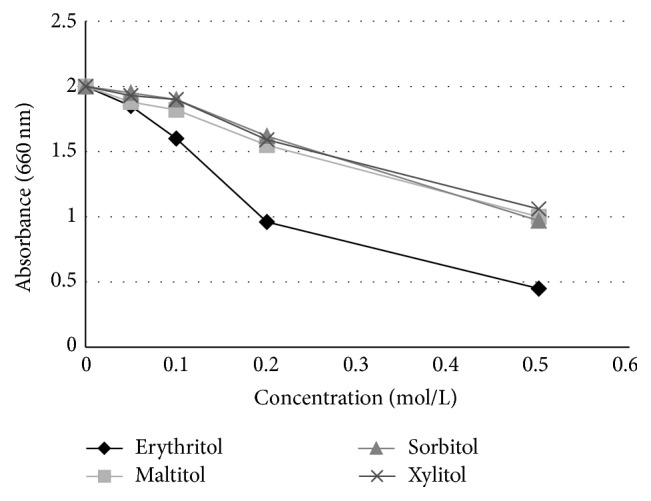
Effect of polyol concentration (mol/L) on growth of* S. mutans* (strain 267-S) after 5 hours. Adapted from Mäkinen et al. [10] and Mäkinen [36].

**Figure 4:**
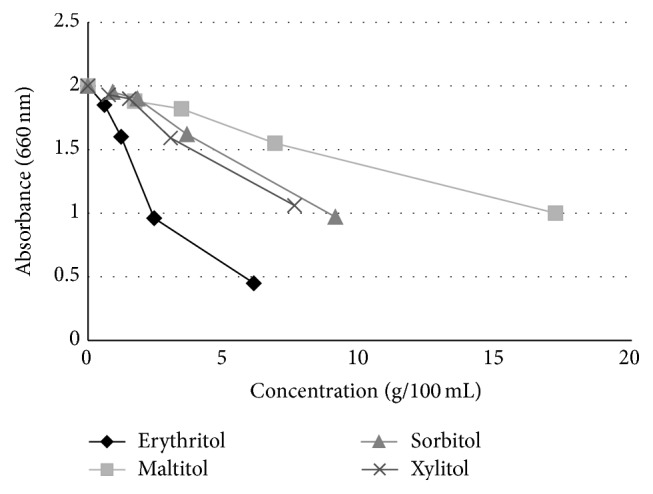
Effect of polyol concentration (g/100 mL) on growth of* S. mutans* (strain 267-S) after 5 hours. Adapted from Mäkinen et al. [10] and Mäkinen [36].

Mäkinen et al. [10] conducted another study that was 3 times longer in duration (6 months) and the daily total polyol consumption was higher (7.0 g/day) with a cohort that was up to 2 times larger (*n* = 30–36) than the previous 2 studies. The teenage subjects (~17 years of age) were given 2 chewable tablets containing xylitol (*n* = 35), erythritol (*n* = 36), or sorbitol (*n* = 36) to suck or chew 6 times daily and underwent examinations (dental evaluations and sample collection of plaque and saliva) prior to polyol exposure (baseline) and at 3 and 6 months. They also were given toothpaste to use containing the corresponding polyol. An untreated control group (*n* = 30) was not given any tablets and was asked to “continue their customary oral hygiene and dietary practice during the study.” The groups showed no statistically significant differences with respect to age or caries experience at the start of the study. The results of this study showed a statistically significant reduction in dental plaque weight as well as a reduction in the levels of* S. mutans* in dental plaque and saliva of subjects using erythritol or xylitol, as shown in Figures 1 and 2, respectively. Notably, the erythritol group not only had a significantly lower plaque weight compared to the control (*p* < 0.05) but also to the sorbitol and xylitol groups (*p* < 0.05) after 6 months. Mäkinen et al. [10] additionally conducted* in vitro* tests with several strains of* S. mutans* in which 0.6 M erythritol, sorbitol, xylitol, or untreated media were incubated with the organism for up to 5 hours. Erythritol inhibited growth most effectively compared with the other polyols (Figures 3 and 4). The results shown in Figure 3 indicate that the effect of xylitol, sorbitol, and maltitol is based on their osmotic effects since the osmolarity (or water activity) is exactly the same for each concentration tested and there is no significant difference in absorbance. The impact of erythritol on growth reduction, however, is higher at the same osmolarity. There is an additional growth-reducing effect of erythritol that xylitol, sorbitol, and maltitol do not have. This may be associated with the ability of erythritol to easily pass the cell membrane passively and suppress growth via several pathways as suggested by Hashino et al. [16] where it interferes in some of the enzymatic pathways involved in the growth of* S. mutans*.  Figure 4 provides a useful insight into how big the differences in growth reduction is between the polyols tested at the same weight/volume concentrations. To reduce the absorbance to, for example, 1, the gram amount of maltitol required to reduce growth to that level is about 7x higher compared to erythritol and for xylitol; it is about 3x higher.

Of interest was the observation that erythritol seemed to inhibit the growth of* S. mutans* by a mechanism that differs from that of xylitol. Normally, xylitol-dependent inhibition of bacterial growth has appeared throughout the entire growth phase, whereas erythritol also inhibited—quite distinctly—the growth of some* S. mutans* strains during later growth phases. Both polyols were considered to have significant utility value in limiting the incidence of dental caries.

In addition to its inhibition of the growth of* Streptococcus*, erythritol was found to decrease the adherence of polysaccharide-forming oral streptococci (14 strains tested:* S. mutans* (9),* S. sanguinis* (2),* S. salivarius* (2), and* S. sobrinus* (1)) in an* in vitro* study investigating the growth inhibition and adherence of cells to a smooth glass surface by 2 or 4% erythritol and xylitol [11]. Both erythritol and xylitol, at a concentration of 4%, significantly reduced the glass surface adhesion of most of the polysaccharide-forming streptococci tested;* S. mutans* 10449 and* S. sobrinus* OMZ 176 were not affected. Growth inhibition was considered not to be associated with the magnitude of the decrease in adherence (for erythritol there was a trend (*p* = 0.12) toward an association) indicating that cell adherence was* via* a mechanism that did not depend on growth inhibition.

In another published study, it was suggested that, when compared to xylitol, erythritol in low concentrations (0.5–2%) had a weaker effect on the bacterial growth and acid production of* S. mutans*, while having stronger effect at high concentrations (8–16%) [12]. White et al. [13] incubated strains of* S. mutans* and* S. sobrinus* with xylitol or erythritol for 48 hours and measured optical density using confocal microscopy at 620 or 640 nm to determine bacterial growth. Both polyols inhibited growth completely: at 15% for erythritol and at 30% for xylitol. No synergistic effect was noted when the polyols were combined.

**Figure 5:**
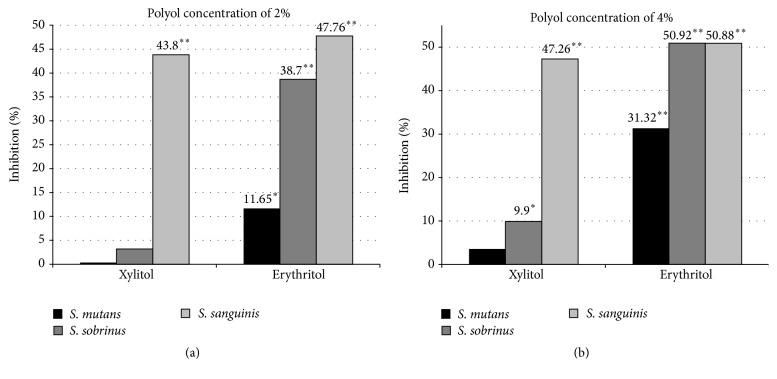
Mean percent inhibition of streptococci biofilm formation by xylitol and erythritol in a microtiter plate assay. From Ghezelbash et al. [14], ^*∗*^ *p* < 0.05 when compared to control using analysis of variance (ANOVA) repeated measures and ^*∗∗*^ *p* < 0.01 when compared to control using analysis of variance (ANOVA) repeated measures (a). From Ghezelbash et al. [14], ^*∗*^ *p* < 0.05 when compared to control using analysis of variance (ANOVA) repeated measures and ^*∗∗*^ *p* < 0.01 when compared to control using analysis of variance (ANOVA) repeated measures (b).

Inhibition of* in vitro* growth and adherence of bacteria and formation of biofilm occurred when 2 or 4% erythritol or xylitol was incubated with streptococci strains (*S. mutans*,* S. sobrinus*, or* S. sanguinis*) overnight in microtiter plates [14]. Erythritol was found to be more effective than xylitol in inhibiting the growth of* S. mutans* (69–71% versus 66–68%). Similar to the findings of Söderling and Hietala-Lenkkeri [11], adherence to the polystyrene microplate, as indicated by 630 nm optical density readings, also was inhibited to a greater extent by erythritol, particularly at the higher concentration of 4%. As shown in Figure 5, erythritol showed a stronger inhibitory effect than xylitol on biofilm formation of all 3 streptococci strains (e.g., 31.32% versus 3.55% inhibition of* S. mutans* at a concentration of 4%). Similar inhibitory effects of erythritol at a concentration of 4% on* S. mutans* were also reported by Saran et al. [15] in 2015 who observed 56.45% and 36.42% inhibition of growth and biofilm formation, respectively.

In an* in vitro* investigation on the effects of polyols on the development of biofilm, Hashino et al. [16] reported that 10% erythritol had an inhibitory effect on the microstructure and metabolomic profiles of biofilm composed of* Porphyromonas gingivalis* and* Streptococcus gordonii*. The most effective reagent to reduce* P. gingivalis* accumulation onto* S. gordonii* substrata was erythritol, when compared with xylitol and sorbitol. The authors suggested that erythritol's inhibitory effects function “*via* several pathways, including suppression of growth resulting from DNA and RNA depletion, attenuated extracellular matrix production, and alterations of dipeptide acquisition and amino acid metabolism.”

**Figure 6:**
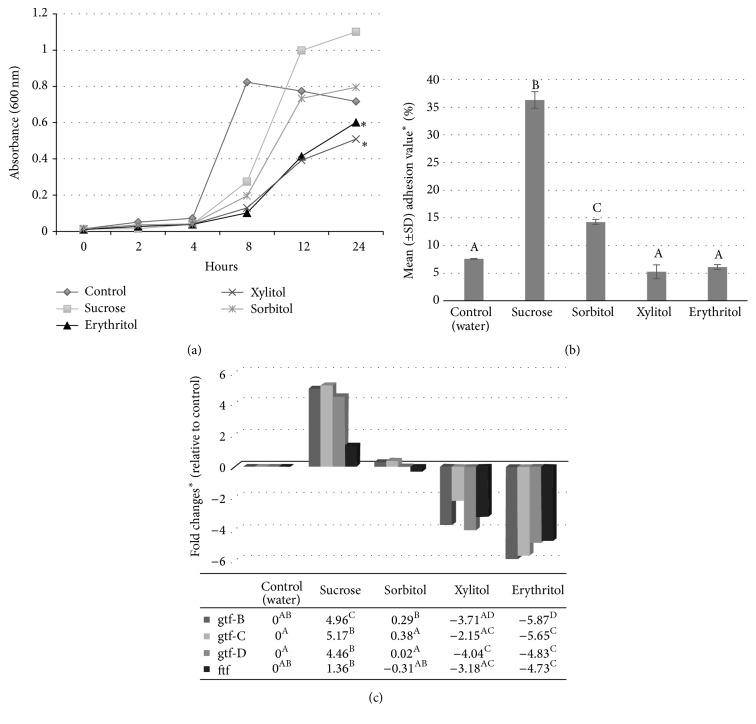
Growth (a), adhesion (b), and gene expression ((c);* gtf* and* ftf*) of* S. mutans *(strain ATCC 31989) in the presence of 10% sucrose, erythritol, xylitol, or sorbitol (adapted from Park et al. [17]). *p* < 0.05 when compared to control (water) using analysis of variance (ANOVA) repeated measures (a). SD: standard deviation; overall difference (*p* < 0.05) based on the Kruskal-Wallis test. ^A, B, C, D^The same letter indicates no significant difference (*p* < 0.05) based on Mann-Whitney testing (b). ^*∗*^ *p* < 0.05, based on the Kruskal-Wallis test, ^A, B, C, D^the same letter indicates no significant difference based on Mann-Whitney testing (c).

The mechanism by which erythritol inhibits growth and reduces adhesion of* S. mutans* was examined by Park et al. [17] through the evaluation of expression profiles of the glucosyltransferase (GTF) and fructosyltransferase (FTF) genes in* S. mutans* in the presence of erythritol. These genes are involved in sucrose metabolism by facilitating the polymerization of free glucose and fructose into glucans and fructans, respectively, which, in turn, act as an energy source and protective barrier against bacterial toxins and are involved in promoting adhesion of bacteria to dental surfaces. In addition, the ability of erythritol to affect adhesion of* S. mutans* to smooth surfaces (i.e., glass beads) was investigated. Initially, the growth of* S. mutans* was evaluated over 24 hours in the presence of 10% sucrose, erythritol, xylitol, sorbitol, or untreated control and showed that both erythritol and xylitol significantly (*p* < 0.05) inhibited growth at a similar level (see Figure 6(a)). Adhesion values and the adhesion inhibition rate of* S. mutans* were significantly (*p* < 0.05) reduced with erythritol and xylitol when compared with sucrose, but not control (water) or sorbitol (see Figure 6(b)). Erythritol and xylitol both significantly decreased the expression of 3 GTF genes and 1 FTF gene (*p* < 0.05) compared to sucrose. The decreases seen with erythritol also were significantly decreased when compared with sorbitol and untreated control (see Figure 6(c)). Since erythritol inhibited the growth of* S. mutans*, reduced adhesion of* S. mutans* to smooth surfaces, and decreased the expression of genes involved in sucrose metabolism, the authors considered erythritol to have anticariogenic potential.

## 5. Erythritol Reduces the Risk of Dental Caries

By the beginning of the new millennium, preliminary information on the oral biology of common polyols had reached a stage that encouraged the undertaking of long-term clinical trials to investigate the effect of erythritol on the incidence and propagation of dental caries in humans. The first, and for the time being the only, long-term human caries trial using erythritol alone (no mixture with other polyols) was executed by Tartu University Institute of Stomatology in Tartu, Estonia, in 2008–2011 [37].

This study resulted from theoretical considerations that the relative effect of three common polyols (i.e., erythritol, xylitol, and sorbitol) on the incidence and propagation of dental caries should differ and reflect the number of hydroxyl groups present in the polyol molecules [38]. Preliminary results obtained with erythritol in animal caries [7] and some oral biologic processes in humans [8–10] were encouraging and, therefore, the Tartu polyol double-blind randomized controlled prospective intervention trial was developed to compare the long-term usage of erythritol and xylitol candies with sorbitol candies in children.

At the start of the trial, 485 first and second grade school children (~8-9 years of age) from 10 schools were randomly divided into erythritol (*n* = 165), xylitol (*n* = 156), and sorbitol (control, *n* = 164) groups. By the end of the 3-year trial, 374 children remained in the study [37]. Those leaving the trial were not at school on examination days, changed schools, or did not wish to continue to participate in the study.

Teachers provided the children with four small chewable tablets containing erythritol, xylitol, or sorbitol to consume three times each school day (about 200 school days per year) resulting in a calculated daily polyol consumption level of about 7.5 g. The children were educated on oral hygiene and provided with a toothbrush and fluoride toothpaste every 6 months with a recommendation to brush their teeth more than once a day. For assessment, the children were assigned to one of 4 trained dental examiners and underwent double-blind clinical examinations at the start of the trial (baseline) and at 12, 24, and 36 months using the International Caries Detection and Assessment System (ICDAS II) [39].

**Figure 7:**
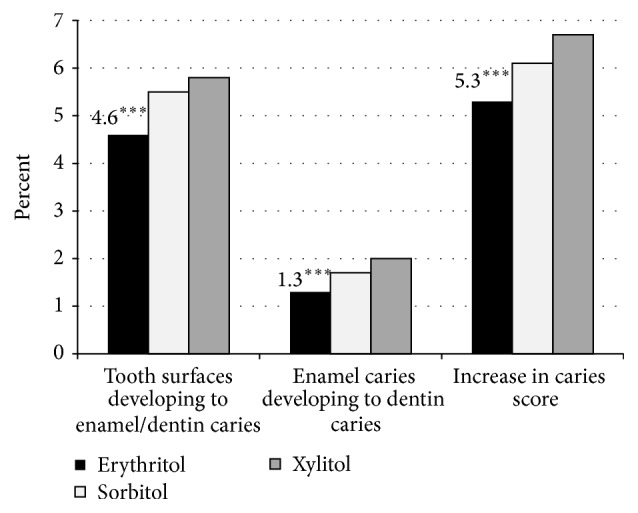
Percentage of tooth surfaces developing into enamel or dentin caries, percentage of enamel caries developing into dentin caries, and percentage of surfaces with an increase in caries score (increase in caries score is transition from any caries score to increase in score of 1 or more) over a 3-year period in a child cohort consuming erythritol-, sorbitol-, or xylitol-containing candies. From Honkala et al. [37]. ^*∗∗∗*^ *p* < 0.001 when compared to sorbitol using Fisher's exact test (two-tailed).

At baseline and at 12 months, the caries indicators of the mixed dentition were similar among all groups. The ICDAS examinations showed that the number of dentin caries teeth and surfaces at the 24-month follow-up and the tooth surfaces at the 36-month follow-up was significantly lower in the mixed dentition in the erythritol group than in the xylitol group. Over the 3-year follow-up period, the erythritol group had significantly less tooth surfaces developing into enamel or dentin caries and significantly less enamel caries tooth surfaces developing into dentin caries when compared with sorbitol and xylitol (see Figure 7). Furthermore, the time of enamel or dentin caries lesions to develop and dentin caries to progress was significantly longer in the erythritol group compared with the other polyol groups. Taken together, this resulted in the erythritol group having 143 less dental treatments (tooth restorations by a dentist) as compared to control.

**Figure 8:**
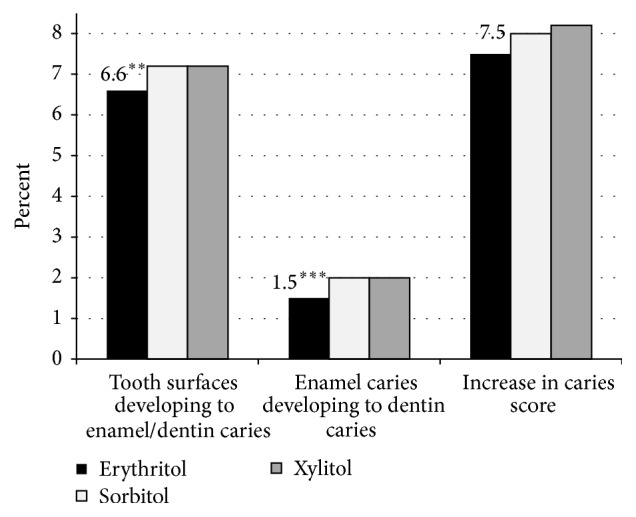
Percentage of tooth surfaces developing into enamel or dentin caries, percentage of enamel caries developing into dentin caries, and percentage of surfaces with an increase in caries score (increase in caries score is transition from any caries score to increase in score of 1 or more) in a child cohort consuming erythritol-, sorbitol-, or xylitol-containing candies 3 years after intervention. From Falony et al. (manuscript submitted). ^*∗∗*^ *p* < 0.05 when compared to sorbitol using Fisher's exact test (two-tailed). ^*∗∗∗*^ *p* < 0.001 when compared to sorbitol using Fisher's exact test (two-tailed).

In 2014, 3 years following cessation of the polyol interventions, 364 of the children were reevaluated using the same procedures (ICDAS examination) used during the 3-year intervention study (Falony et al., manuscript submitted). No significant differences in decayed, missing, and filled teeth and surfaces between the intervention groups were noted; however, in the erythritol group, percentages of surfaces developing enamel/dentin caries or dentin caries or subject to dentist intervention were still reduced compared to the other groups (see Figure 8). Consequently, habitual usage of erythritol candies in this child cohort showed a slower and lower caries development compared to the xylitol and sorbitol groups.

**Figure 9:**
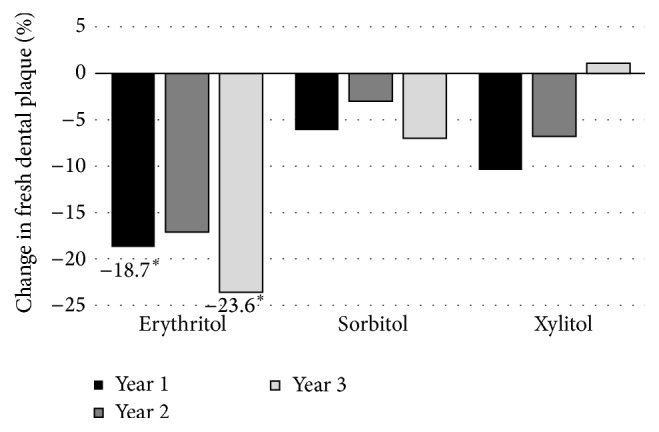
Percent change in dental plaque weight against baseline over a 3-year period in a child cohort consuming erythritol-, sorbitol-, or xylitol-containing candies. Adapted from Runnel et al. [40]. ^*∗*^ *p* < 0.05 when compared to baseline using the Wilcoxon Signed Rank test with the Bonferroni correction.

As part of this study, but published separately [40], saliva and plaque were collected at time of dental examination for determination of salivary and plaque counts of* S. mutans* and salivary counts of* Lactobacillus*. At years 1 and 3, a significant reduction (*p* < 0.05 when compared with the baseline values) in the weight of freshly collected dental plaque of the subjects occurred in the group receiving erythritol with a reduction tendency at the 2-year examination, as shown in Figure 9. No such changes were found in groups receiving sorbitol or xylitol. Chemical analysis indicated that usage of the three polyols had no significant or consistent effect on the plaque levels of protein, glucose, glycerol, or calcium. However, after three years, the plaque of erythritol-receiving subjects contained significantly (*p* ≤ 0.05) smaller levels of acetic acid and propionic acid than that of subjects who had received xylitol or sorbitol. The plaque levels of lactic acid partly followed this same general pattern. The consumption of erythritol was also generally associated with significantly (*p* < 0.05) lower counts of salivary and plaque* S. mutans*. The use of these polyols had no significant effect on salivary* Lactobacillus* levels. Three months after the end of the trial, a fourth group of children (*n* = 162) was evaluated as an additional comparison group within the same age groups. In this comparison group, mean salivary* S. mutans* counts were significantly higher than in the erythritol and xylitol groups (*p* = 0.014 and 0.034, resp.), but not the sorbitol group. Taken together, these results suggested that habitual consumption of erythritol reduced the involvement of several oral biologic factors that have normally been associated with the initiation and propagation of dental caries. Consequently, these results were in congruence with the clinical caries observations reported by Honkala et al. [37].

With regard to erythritol, the anticariogenic results of the Honkala et al. [37] study were consistent with previous findings in experimental animals and human subjects. The xylitol results, however, were not so straightforward and did not show as strong of an effect as erythritol when compared with sorbitol. Few previous xylitol caries studies [42–45] used sorbitol as a control and those results did not consistently show that xylitol outperformed sorbitol. In terms of demonstrable effectiveness, there are some inconsistencies in the data between xylitol and sorbitol, making the determination of the more effective compound less clear. For this reason, both xylitol and sorbitol were commonly used to compare to erythritol, which consistently outperformed both.

It may also be possible that the consumption level of the polyols and especially the frequency of daily use of the saliva stimulants (only three teacher-supervised exposures) were too low in this child population and under the prevailing school-based program conditions to show a difference for xylitol versus sorbitol. Furthermore, (1) the polyol tablets had to be consumed at schools within a relatively short duration of the day, as stipulated by the school hours (children left school latest at 2 pm); (2) chewing gum used in most xylitol studies is a much better salivary flow stimulant [46] compared to the compressed tablets used in the Honkala et al. [37] study, which used tablets purposely to focus as much as possible on the pharmacological effects of the polyols; (3) the exposure to the school children was a very mild intervention that did not take place during weekends and vacations meaning that intervention was only for about 200 days per year; and (4) erythritol has a lower solubility and molecular weight than xylitol and, therefore, erythritol dissolves more slowly most likely resulting in longer exposure and diffuses faster and deeper into the dental plaque where it can better exercise its impact on microbes like* S. mutans*.

The results from Honkala et al. [37], Runnel et al. [40], and Falony et al. (manuscript submitted) investigations indicate that erythritol did show statistically significant differences from the other two polyols in terms of caries development and oral biologic processes. In these studies, erythritol turned out to be a potential caries-preventing dietary sucrose substitute with higher efficacy compared to sorbitol and xylitol.

Some reports have claimed nonefficaciousness of erythritol as a caries-limiting agent. Closer examination of the study designs involved has revealed serious generalizations and shortcomings. For example, in a review of a double-blind, cluster-randomized clinical trial in school children (~10 years of age) conducted by another group of researchers [41], Duane [47] commented that there was no evidence of caries reduction in a school xylitol and erythritol lozenge program. However, the overall length of the intervention period may have been too short (±190 intervention days in 9 months or ±380 intervention days in 21 months), while the frequency of use (3x per school day) and the amount of xylitol (4.7 g) and erythritol (4.5 g) seemed to be too low. Furthermore, final caries diagnoses were made 27 or 39 months after termination of the interventions, and the study subjects lived in a fluoridated area and exhibited low caries activity.

The clinical studies are summarized in Table 2.

## 6. Erythritol Supports Periodontal Therapy

Traditional subgingival root scaling using hand tools is considered “technically demanding” and “time consuming” and, if not done carefully, could lead to painful dental root tissue loss [48–52]. Air-polishing treatment with nonabrasive powders such as glycine powder can reduce tissue loss on root surfaces while causing less pain for patients [53]. Since erythritol has similar abrasive properties and particle size to glycine and has a sweet taste and noncariogenic properties, it was studied for its potential use with air-polishing devices and compared with traditional root scaling methods [49].

In a randomized, controlled, parallel-group clinical trial, subjects underwent supportive periodontal therapy at the start of the trial (baseline) and after 3 months [49]. Out of 39 subjects, there were 91 and 87 periodontal sites used in the test and control groups, respectively. In the erythritol test group, test sites were subgingivally treated for 5 seconds with an air-polishing device using erythritol. An experienced operator treated control sites with curettes until all subgingival deposits were removed. All remaining dentition was treated using standard supportive periodontal therapy. Based on a visual analog scale, patient's tolerance was significantly better for sites treated with erythritol than those without. There were no differences in clinical outcomes between subgingival air-polishing with erythritol or traditional scaling except that patients tended to prefer air-polishing. More recently, Hägi et al. [54] published the results of a similar study, but over a period of 6 months, in which subjects underwent treatment at baseline and 3 and 6 months with subgingival low abrasive erythritol powder using an air-polishing device or repeated scaling and root planing at study sites identified at baseline as bleeding on probing positive sites with probing pocket depth of ≥0.4 mm but no detectable calculus. At baseline and 6 months, plaque index, bleeding on probing, probing pocket depth, clinical attachment level, and subgingival plaque were evaluated. In the 38 patients completing the study, both treatments produced significant reductions in bleeding on probing and probing pocket depth and increases in clinical attachment level. There were no statistically significant differences between the treatment groups.

In another study, subgingival air-polishing with erythritol containing 0.3% chlorhexidine was compared to ultrasonic debridement at 3-month intervals for up to 12 months [55]. Fifty patients with 6,918 sites were examined at start of the study (baseline) and served as their own controls (i.e., one side was treated with erythritol air-polishing and one side with ultrasonic debridement). At the 12-month examination, there was no difference between the treatments with respect to the presence or absence of a probing depth >4 mm and the frequencies at >1,000 and >100,000 cell/mL of 6 microorganisms (*Porphyromonas gingivalis, Aggregatibacter actinomycetemcomitans, Tannerella forsythia, Treponema denticola, Prevotella intermedia,* and* Parvimonas micra*). However, at 12 months, erythritol-treated sites were less frequently positive for* Aggregatibacter actinomycetemcomitans* at >1,000 cell/mL with counts never exceeding 100,000 cells/mL. Moreover, air-polishing with erythritol was significantly better than ultrasonic debridement in terms of pain/discomfort perception.

An* in vitro* study was conducted to compare the efficacy of air-polishing with 99.7% erythritol/0.3% chlorhexidine versus standard glycine powder and their antimicrobial and antibiofilm potential on* Staphylococcus aureus, Bacteroides fragilis*, and* Candida albicans* [56]. For each strain, 6 sandblasted titanium disks were used: 2 for air-polishing with water, 2 for air-polishing with glycine, and 2 for air-polishing with erythritol/chlorhexidine. The amount of biofilm was determined by spectrophotometric assay and biofilm residue was examined for microbial recovery. Erythritol/chlorhexidine was significantly more effective than glycine in inhibiting the growth of all 3 strains, reducing the number of surviving cells following air-polishing (15–30% for glycine, 50% for erythritol/chlorhexidine), and reducing the biofilm produced by all 3 strains.

The air-polishing studies are summarized in Table 3.

## 7. Discussion

Erythritol is the newest polyol (sugar alcohol) used as a bulk sweetener in foods. It differs in many ways from all other polyols. It has the smallest molecular size as this polyol is of the tetritol type and it is the first polyol to be commercially produced by fermentation, a natural process [1]. Its unique metabolic profile renders it to be noncaloric, nonglycemic, noninsulinemic, and very well tolerated. It has been consumed by animals and humans for ages as small quantities of erythritol occur widely in microorganisms, algae, fermented foods, lichens, mushrooms, many fruits and vegetables, and also animal and human tissues [5, 20, 57].

Unlike all other polyols including sorbitol and xylitol, ingested erythritol is rapidly and almost completely absorbed from the small intestine, not metabolized, and excreted unchanged in the urine [32]. Depending on the quantity ingested, approximately 10% of ingested erythritol may reach the colon [5]. Its high systemic bioavailability has been linked to additional health benefits for people with diabetes by reducing arterial stiffness and improving small vessel endothelial function [18].

Owing to its sweet taste and high digestive tolerance, erythritol is well suited to replace sugar pound-for-pound in foods without replacing any calories thereby significantly reducing the energy density of those foods. All dental and oral biological studies carried out to date have suggested erythritol to be noncariogenic. Erythritol is being used as a sweetener in dentally safe confectionery items, desserts, tabletop sweeteners, beverages, and many other sugar-free and calorie-reduced foods. Erythritol is authorized for use in foods in more than sixty countries and is included in the GSFA-list (General Standard for Food Additives) of the* Codex Alimentarius* under INS number 968.

The noncariogenicity of erythritol was first investigated and established in rats in 1990 [7, 58] and soon after in 1996 in humans [59]. Early studies demonstrated that erythritol limits the growth, lactic acid production, and plaque formation of* S. mutans* (serotypes a–h) [58] and a number of other streptococci species [10, 14]. Erythritol did not serve as a substrate for cellular aggregation of* S. mutans* (serotypes d, g, and h) and was not utilized for water-insoluble glucan synthesis and cellular adherence by glucosyltransferase from* S. mutans* PS-14 (c) or* S. sobrinus* 6715 (g) [7]. Moreover, erythritol decreased the adherence of polysaccharide-forming oral streptococci when present in growth media at levels as low as 2–4% [11, 14]. Most of the* in vitro* studies compared the impact of erythritol on inhibition of microbial growth and adhesion with one or more other polyols with mostly similar inhibitory effects when compared to xylitol and no effects for sorbitol. This was consistent with the findings in a 6-month human study investigating the effects of candies formulated with erythritol, xylitol, and sorbitol on premonitory symptoms of dental caries [10]. In this study, the only difference in beneficial effects seen for erythritol and xylitol but not for sorbitol was a significant higher reduction in fresh plaque weight compared to baseline in subjects consuming erythritol (−30%) than those consuming xylitol (−13%). Such higher dental plaque reduction for erythritol versus xylitol was also seen in a 3-year caries study in 485 children comparing the effect of candies formulated with erythritol, xylitol, and sorbitol: a reduction in dental plaque was only demonstrated in the group of children consuming erythritol candies (−24%) but not in the xylitol or sorbitol group [40]. This caries study further demonstrated a lower caries development in the erythritol group compared to the sorbitol and xylitol group after a 3-year intervention period [37] that was still visible even 3 years after the intervention was terminated (Falony et al., manuscript submitted). The results reported in these three publications showed that erythritol has caries-preventing activity with higher efficacy compared to sorbitol and xylitol. Broadly, the mechanisms through which the caries-preventing activity of erythritol is achieved are as follows:

- Inhibition in growth and decreased acid production of the principle bacterial species associated with caries development like* S. mutans*.
- Decrease in adherence of common streptococcal oral bacteria to tooth surfaces, due, in part, to a decrease in expression of bacterial genes involved in sugar(s) metabolism resulting in a reduced production of glucans and fructans.
- Decrease in* in vitro* biofilm formation and* in vivo* dental plaque weight.

Certain exposure conditions should be met in order to benefit from the caries-preventing activity of erythritol. The two short term studies by Mäkinen et al. in 2001 and 2002 [8, 9] that did not show plaque reduction used an intervention period of 2 months and used very fragile tablets that fragmented and dissolved in the mouth rapidly resulting in an exposure time of 1 to 2 minutes. The total daily dose of erythritol in both studies was 5.1 g and 2.7 g in 2001 and 2002, respectively. In addition, these tablets contained low use levels of erythritol: 39.5% and 24.5% in 2001 and 2002, respectively. The short exposure time combined with the rather low use level and low total daily dose of erythritol, as well as the short intervention period of 2 months, has likely contributed to the absence of a plaque reducing effect in these two studies. The caries study by Hietala-Lenkkeri et al. 2012 [41] that did not show caries-preventive effects of erythritol and xylitol used lozenges containing 49.4% erythritol that were consumed 3 times daily resulting in a total dose of 4.5 g erythritol daily. The actual intervention duration was 9 and 18 months in the 2 groups investigated who were, respectively, examined 39 and 27 months after termination of the intervention. In addition, the study was done in an area with low caries prevalence: average DMFT for 12-year-olds was 0.8 in comparison with the average for Finland which is 1.2. This, and the rather low use level and low total daily dose of erythritol, as well as the short intervention period of maximally 18 months and the absence of a clinical examination immediately after the intervention, hs likely contributed to the absence of a caries reducing effect in this study.

The erythritol candies used in the human studies published by Mäkinen et al. [10], Runnel et al. [40], Honkala et al. [37], and Falony et al. (manuscript submitted) were conducted with pressed tablets/lozenges containing 90% erythritol and with a hardness similar to such candies on the market that take about 4 to 8 minutes of sucking time to dissolve in the mouth [46, 60]. In particular, the beneficial results from the 3-year caries study reported in the last 3 publications were achieved under the following exposure conditions:

1. Candies containing 90% erythritol.
2. Daily consumption of 7.5 g erythritol, divided over three consumptions of 2.5 g erythritol.
3. Candies with a hard texture resulting in an exposure time of about 4 minutes or more per eating occasion.
4. Consumption only during schooldays, so not during weekends or summer holiday (about 200 days per year).

These exposure conditions are much in line with recommendations for xylitol chewing gum as published in the 2011 review by Mäkinen [36].

## 8. Conclusions

The present review summarizes the oral health benefits of erythritol use as demonstrated by a reduction in the overall number of dental caries and associated dental surface restorations (dentist treatments) when used routinely. It also can serve as a suitable matrix for subgingival air-polishing to replace traditional root scaling in periodontal therapy. The dental and oral biological studies on erythritol, xylitol, and sorbitol discussed have reemphasized important differences between the individual polyols. Polyols can therefore not be regarded as a single entity of organic molecules with exactly identical molecular parameters and similar biological effects. The evidence demonstrating better efficacy of erythritol compared to sorbitol and xylitol to maintain and improve oral health is growing and offers a clear distinction among polyols.

## Figures and Tables

**Table 1:** Summary of *in vitro* and *in vivo* bacterial (dental plaque) growth inhibition studies with erythritol.

Study type	Substance tested	Subjects or strain	*n*	Age (years)	Dose or concentration	Results	Reference
Randomized, double-blinded clinical study	Erythritol or xylitol	Healthy adults and physically or mentally disabled adults	15	~30	5.2 g/day, 5x/day for 2 months (tablets)	Xylitol, but not erythritol, showed a statistically significant reduction of dental plaque and saliva and plaque levels of *S. mutans*	Mäkinen et al. (2001) [8]

Clinical study	Erythritol/xylitol, sorbitol/xylitol, xylitol, or sorbitol	Mentally disabled adults	22–26	~30	5.4 g/day (2.7 g of each polyol), 5x/day for 64 days (tablets)	A significant reduction in plaque and saliva counts of *S. mutans* was demonstrated for xylitol alone and for the 1 : 1 xylitol mixture with erythritol; the relative portion of *S. mutans* of total streptococci was significantly higher in the sorbitol group compared with the sorbitol-erythritol group	Mäkinen et al. (2002) [9]

Clinical study	Erythritol, xylitol, or sorbitol	Healthy teenagers	35-36	~17	7 g/day, 6x/day (tablets) plus 2x/day (toothpaste)	Significant reduction in the levels of *S. mutans* in dental plaque and saliva with erythritol or xylitol; only the erythritol group had significantly lower plaque weight compared to the control, sorbitol and xylitol groups	Mäkinen et al. (2005) [10]

*In vitro* growth inhibition	Erythritol, xylitol, or sorbitol	*S. mutans*	—	—	0.6 M for 5 hours	Erythritol inhibited growth “most effectively” compared with the other sugar alcohols	Mäkinen et al. (2005) [10]

*In vitro* glass adhesion and growth inhibition	Erythritol or xylitol	*S. mutans*, *S. sanguinis*, *S. salivarius*, and *S. sobrinus*	—	—	2 or 4%	Erythritol and xylitol, at 4%, significantly reduced the glass surface adhesion Growth inhibition was not associated with the magnitude of the decrease in adherence	Söderling and Hietala-Lenkkeri (2010) [11]

*In vitro* growth inhibition and acid production	Erythritol or xylitol	*S. mutans*	—	—	0.5–16%	Compared to xylitol, erythritol in low concentrations (0.5–2%) had a weaker effect on the bacterial growth and acid production of *S. mutans*, while having stronger effect at high concentrations (8–16%)	Yao et al. (2009) [12]

*In vitro* growth inhibition	Erythritol or xylitol	*S. mutans* and *S. sobrinus*	—	—	2.35–300 mg/mL	Erythritol (at 150 mg/mL) and xylitol (at 300 mg/mL) inhibited growth	White et al. (2015) [13]

*In vitro* polystyrene plate adhesion, biofilm formation, and growth inhibition	Erythritol or xylitol	*S. mutans*, *S. sanguinis*, and *S. sobrinus*	—	—	2 or 4%	Erythritol more effective than xylitol in inhibiting growth, adherence, and biofilm formation	Ghezelbash et al. (2012) [14]

*In vitro* growth inhibition and reduction in biofilm formation	Erythritol	*S. mutans*	—	—	0.5–10%	Erythritol significantly inhibited growth (>78%) and biofilm formation (40.2%)	Saran et al. (2015) [15]

*In vitro* biofilm formation	Erythritol, xylitol, or sorbitol	*Porphyromonas gingivalis* and *S. gordonii*	—	—	0.8, 5, or 10%	Most effective reagent to reduce *P. gingivalis* accumulation onto *S. gordonii* substrata was erythritol when compared with xylitol and sorbitol; erythritol suppressed the endopeptidase, Rgp.	Hashino et al. (2013) [16]

*In vitro* growth inhibition mechanism by expression of GTF and FTF genes	Erythritol, xylitol, sucrose, or sorbitol	*S. mutans*	—	—	10%	Erythritol and xylitol significantly inhibited growth at a similar level and decreased the expression of 3 GTF genes and 1 FTF gene compared to sucrose; the gene expression decreases seen with erythritol also were significantly decreased when compared with sorbitol and untreated control; adhesion values and the adhesion inhibition rate were significantly reduced with erythritol and xylitol when compared with sucrose, but not control (water) or sorbitol	Park et al. (2014) [17]

GTF: glucosyltransferase; FTF: fructosyltransferase.

**Table 2:** Summary of dental caries clinical trials with erythritol.

Study type	Substance tested	Subjects	*n*	Age (years)	Dose	Results	Reference
Double-blind randomized controlled prospective intervention trial	Erythritol, xylitol, or sorbitol	School children	156–165	~8-9	7.5 g/day, 3x/school day (~200 school days/year) for 3 years	Erythritol group had significantly less tooth surfaces developing into enamel or dentin caries and significantly less enamel caries tooth surfaces developing into dentin caries when compared with sorbitol and xylitol; time of enamel or dentin caries lesions to develop and dentin caries to progress were significantly longer with erythritol	Honkala et al. (2014) [37]

Double-blind randomized controlled prospective intervention trial (Examinations 3 years after cessation of all interventions in Honkala et al. (2014) study)	Erythritol, xylitol, or sorbitol	School children	129	~14-15	7.5 g/day, 3x/school day (~200 school days/year) for 3 years followed by 3 years without any intervention	No significant differences in decayed, missing, and filled teeth and surfaces between the intervention groups were noted However, erythritol group still had reduced percentages of surfaces developing enamel/dentin caries, dentin caries, or subject to dentist intervention compared to other groups	Falony et al. (manuscript submitted)

Salivary and plaque counts of *S. mutans* and salivary counts of *Lactobacillus* (Sampling from Honkala et al. (2014) study)	Erythritol, xylitol, or sorbitol	School children	156–165	~8-9	7.5 g/day, 3x/school day (~200 school days/year) for 3 years (tablets)	At years 1 and 3, a significant reduction in the weight of freshly collected dental plaque of the subjects occurred with erythritol No such changes with sorbitol or xylitol; no effect on the plaque levels of protein, glucose, glycerol, or calcium; erythritol was also generally associated with significantly lower counts of salivary and plaque *S. mutans* No effect on salivary *Lactobacillus* levels	Runnel et al. (2013) [40]

Cluster-randomized, double-blinded clinical trial	Erythritol/maltitol or xylitol/maltitol	Healthy children	96–101	~10	4.5 g erythritol + 4.2 g maltitol/day 4.7 g xylitol + 4.6 g maltitol/day, 3x/day for up to 2 years with a 4-year follow-up (lozenges)	No evidence of caries reduction; however, final caries diagnoses were made 27 or 39 months after termination of the interventions, and the study subjects lived in a fluoridated area and exhibited low caries activity	Hietala-Lenkkeri et al. (2012) [41]

**Table 3:** Summary of periodontal studies on air-polishing with erythritol.

Study type	Substance tested	Subjects or strain	*n*	Age (years)	Treatment	Results	Reference
Randomized, controlled, parallel-group 3-month clinical trial	Erythritol	Adults	39	~55	Subgingivally treated for 5 seconds with an air-polishing device using erythritol or standard supportive periodontal therapy	No differences in clinical outcomes between subgingival air-polishing with erythritol or traditional scaling except that patients tended to prefer air-polishing with erythritol	Hägi et al. (2013) [49]

Randomized, controlled, parallel-group 6-month clinical trial	Erythritol	Adults	38	~55	Subgingival low abrasive erythritol powder using an air-polishing device or repeated scaling and root planing at study sites	Both treatments produced significant reductions in bleeding on probing and probing pocket depth and increases in clinical attachment level; no statistically significant differences between the treatment groups	Hägi et al. (2015) [54]

Randomized clinical trial	Erythritol	Adults	50	~8-9	Subgingival air-polishing with erythritol containing 0.3% chlorhexidine was compared to ultrasonic debridement at 3-month intervals for up to 12 months	No difference between the treatments with respect to the presence or absence of a probing depth and the frequencies of 6 microorganisms; erythritol-treated sites were less frequently positive for *Aggregatibacter actinomycetemcomitans *Air-polishing with erythritol was significantly better than ultrasonic debridement in terms of pain/discomfort perception	Müller et al.(2014) [55]

*In vitro* antimicrobial and antibiofilm study using sandblasted titanium disks	Erythritol	*Staphylococcus aureus, Bacteroides fragilis*, and *Candida albicans*	2	—	Air-polishing with 99.7% erythritol/0.3% chlorhexidine versus standard glycine powder	Erythritol/chlorhexidine was significantly more effective than glycine in inhibiting the growth of all 3 strains, reducing the number of surviving cells following air-polishing (15–30% for glycine, 50% for erythritol/chlorhexidine) and reducing the biofilm produced by all 3 strains	Drago et al. (2014) [56]
